# Quantification of quercetin from red onion (*Allium cepa* L.) powder via high‐performance liquid chromatography‐ultraviolet (HPLC‐UV) and its effect on hyperuricemia in male healthy Wistar albino rats

**DOI:** 10.1002/fsn3.3822

**Published:** 2023-11-22

**Authors:** Muhammad Umer, Mahr Un Nisa, Nazir Ahmad, Muhammad Abdul Rahim, Ladislaus Manaku Kasankala

**Affiliations:** ^1^ Department of Nutritional Sciences, Faculty of Medical Sciences Government College University Faisalabad Punjab Pakistan; ^2^ Department of Food Science, Faculty of Life Sciences Government College University Faisalabad Punjab Pakistan; ^3^ Department of Food Science and Nutrition Tanzania Food and Nutrition Centre Dar es Salaam Tanzania

**Keywords:** biochemical parameters, growth performance, HPLC‐UV, nitrogen balance, nutrient digestibility, onion (*Allium cepa* L.), quercetin

## Abstract

Onions (*Allium cepa* L.) contain various flavonols, including quercetin, kaempferol, anthocyanin, luteolin, and myricetin. Quercetin in onions is considered the primary bioactive component. To assess the impact of quercetin on hyperuricemia in healthy Wistar albino rats, this study used high‐performance liquid chromatography with ultraviolet (HPLC‐UV) to identify and measure quercetin in onion powder. Twenty‐four 160 ± 10 g, six wistar albino male rats in each group were kept: NC (control sample, no onion powder), OT_1_, OT_2_, and OT_3_, which contained 11.13, 14.84, and 18.61 g/100 g onion powder, respectively. The treatment lasted 28 days, during which the last 7 days were for urine, feces, and blood collection. The results showed a trend of decreasing levels of alanine aminotransferase (ALT), aspartate aminotransferase (AST), alkaline phosphatase, total bilirubin, total cholesterol, and low‐density lipoprotein in rats fed OT_1_, OT_2_, and OT_3_ diets. Improvements were observed in feed, water, and nutrient intake, feed conversion ratio, feed efficiency ratio, nutrient digestibility, nitrogen balance, body weight, blood urea nitrogen, creatinine, and uric acid levels (*p* ≤ .05). In contrast, high‐density lipoprotein, triglycerides, serum total protein, neutrophils, and lymphocytes did not change (*p* ≥ .05). White blood cells, red blood cell count, platelet count, hemoglobin, and monocytes showed an upward trend. Based on our calculations, we determined the optimal human dosage from the most effective amount of onion powder. By taking into account the ratio of human‐to‐rat surface area, we estimate that the equivalent human dose of onion is 181.04 grams with 204 mg of quercetin. Additionally, when factoring in the dry matter content, the recommended dose of onion is 29.19 grams with 220 mg of quercetin.

## Graphical abstract

This study used high‐performance liquid chromatography with ultraviolet (HPLC‐UV) to identify and measure quercetin in onion powder and investigate its effect on hyperuricemia in healthy Wistar albino rats. From the results, using the human‐to‐rat surface area ratio, the estimated corresponding human dose of onion with 204 mg of quercetin was 181.04 grams. The recommended daily consumption of onion was 29.19 grams with 220 mg of quercetin based on dry matter content.

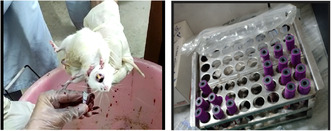

## 1 INTRODUCTION

Hyperuricemia is a medical condition that occurs when the body consumes meals that are high in protein and purines, along with high fructose, has less physical activity, and is overweight (Slimestad et al., 2007). During the winter season, hyperuricemia might be high in the serum due to less water intake (Zuo et al., 2016). This imbalance in nutrient intake can lead to inflammation, gout, hypertension, hyperlipidemia, urolithiasis, and renal insufficiency (Edwards, 2009; Mazzali et al., 2001). In Pakistan, the prevalence of hyperuricemia has increased by 25.3% (Qudwai & Jawaid, 2017). However, onions contain phenolic compounds such as quercetin, kaemferol, and isorhamnetin in the form of flavonols that can reduce the risk of hyperuricemia. These compounds can inhibit the production of uric acid in the liver by catalyzing the oxidation of hypoxanthine to xanthine and preventing xanthine oxidase (XO) activity. While most research has focused on the effect of quercetin on XO activity, these compounds also have antioxidant and hepatoprotective properties (Candan, 2003; Cos et al., 1998; Griffiths et al., 2002; Haidari et al., 2008; Kukongviriyapan et al., 2003; Mittal et al., 2008; Mo et al., 2007; Nile & Khobragade, 2011). Due to the richness of phenolic compounds, including quercetin, onions have many positive effects on health, such as being an antioxidant, anti‐carcinogenic, antimutagenic, anti‐asthmatic, immune‐enhancing, antibacterial, prebiotic, and xanthine oxidase inhibitory agent. People use fresh onions or onion powder in different dishes, such as salads and sandwiches. However, very limited information is available on the safe level of red onion in the diet. To explore the safe level of onion powder in healthy rats, a study was conducted on healthy male albino rats to determine water and feed intake, nutrient digestibility, nitrogen status, weight management, serum biochemicals, and hematological parameters.

## 2 MATERIALS AND METHODS

### 2.1 Procurement of raw material

Red onions (*Allium cepa* L.) were purchased from a local vegetable market and processed by removing any undesirable particles or peels. All chemicals used in this research were purchased from Sigma‐Aldrich.

### 2.2 Preparation of onion powder and proximate analysis of onion powder

First, the red onion was sliced and placed in a hot air oven set to 65°C for 24 h. After it was thoroughly dried, an electric lab grinder was used to create onion powder. The powder was then placed in a plastic bag and stored in the refrigerator at 4°C for later analysis, as per the method described by Arslan & Özcan, 2010. The nutrient composition of the onion powder was analyzed using the AOAC method of 2006.

### 2.3 Determination of quercetin in onions using HPLC‐UV

#### 2.3.1 Chemicals and preparation of the sample

10 g of the sample were added to the solvent in an 80:20 v/v methanol‐to‐water ratio for extraction. The material was shaken for 4 h at 150 rpm on an orbital shaker. The Whatmann paper was used to filter the extract. Following filtration, the residue was once again extracted using an 80:20 (v/v) methanol: water mixture. By employing a rotary evaporator at 40°C until all of the methanol had been evaporated, the resulting extract was concentrated. In order to do the HPLC‐UV analysis, the final semisolid extract was collected and taken to the hi‐tech lab (Kwak et al., 2017). The Department of Botany at Government College University, Faisalabad, offered the Quercetin (Q) standard.

#### 2.3.2 HPLC‐UV method

Chemicals of HPLC grade were bought from Sigma‐Aldrich (St. Louis, MO, USA). The most recent Agilent HP1200 was made to attach to the G1322A quaternary pump and degasser for the quantification of quercetin in onion metabolic extract. The pattern of the chromatogram was noted using the G1314A wavelength UV detector, and the particle size was set at 5 m (250 4.0 mm) in the ODS column (Lichrospher 100, Merck, Darmstadt, Germany). A 1 mL sample in HPLC‐grade water was added to a 50 mL volume to prepare it. The sample was then filtered using Whatman No.1 (pore size 25 m) and placed in a water bath shaker for 5 min at 30°C. It was then injected into an HPLC system and run with HPLC‐grade water as the mobile phase. The functional components in the extract were observed by comparing them to the reference quercetin and kaempferol, and the results were recorded by maintaining a flow rate of 1 mL/min. Mobile phase A (95% formic acid in water by volume) and mobile phase B (methanol) were used to achieve chromatographic separation. Using 50% mobile phase B for 10 minutes, rising to 80% B over 10 minutes, then to 100% B over 11 min, and then returning to 50% B, linear gradient elution was carried out. UV monitoring was done at 360 nm with the flow rate kept at 1.0 mL/min (Rahim et al., 2022).

### 2.4 Study design and animals

A trial was conducted in collaboration between the Department of Nutritional Sciences and the Department of Physiology at Government College University Faisalabad, Punjab, Pakistan. The trial involved twenty‐four adult, healthy male Wistar albino rats weighing 160 ± 10 g. The rats were housed in an animal laboratory at a temperature of 25 ± 5°C and a humidity of 45%–55%. All animals were treated according to laboratory animal care and use guidelines (Wade, 1978), and the study was approved by the Animal Ethical Committee of Government College University, Faisalabad, Pakistan‐wide Ref. No. GCUF/ERC/85. Onion powder was given to the rats on the base of the quercetin quantity in three levels: 0.075 mg/kg, 0.100 mg/kg, and 0.125 mg/kg. The trial lasted for 42 days, with 14 days of feed adjustment period and 28 days of treatment and collection period (Williams et al., 1984).

### 2.5 Composition of diet and nutrient digestibility

The standard method of AOAC (2006) was used to determine the nutrient composition in both the feed and feces for the determination of nutrient intake and its digestibility (Nisa et al., 2006; Shi et al., 2015). Table 1 shows that vitamins and minerals were added to the diet as per the given formulation. The animals were given *isocaloric* and *isonitrogenous* diets, prepared as per the rodent diet formulation by Reeves et al. (1993).

(1) $$\text{Digestibility}=\frac{\text{Nutrient Intake}-\text{Nutrient in feces}}{\text{Nutrient Intake}}\times 100$$

**TABLE 1:** Composition and nutritive value of diet.

Ingredients	NC	OT_1_	OT_2_	OT_3_
Corn starch	286	286	286	286
Dextrose	286	286	286	286
Corn oil/Soybean oil	70	70	70	70
Soyabean meal	496	496	496	496
AIN‐93‐MX Mineral Mix	35	35	35	35
AIN‐93‐vitamin mix	10	10	10	10
TBHQ (Tert‐butylhydroquinone)	0.008	0.008	0.008	0.008
Onion powder (g)		11.13 g (0.75 mg/kg)	14.84 g (0.100 mg/kg)	18.61 g (0.125 mg/kg)
Total calories (Kcal/kg)	3766	3766	3766	3766
Nutritive value (%)				
Dry matter	66.22	72	70	69.80
Crude protein	17.63	18.63	17.24	16.75
Crude fiber	1.27	1.97	1.52	1.41
Ether extract	6.81	6.13	6.62	6.05
Ash	3.70	3.4	3.17	3.28
Moisture	33.78	28	30	30.2
NFE	36.81	41.87	41.45	42.31

Abbreviations: NC, Normal control; OT_1_, Onion treatment 1; OT_2_, Onion treatment 2; OT_3_, Onion treatment 3.

### 2.6 Evaluation of weight change and FCR and FER

The feed conversion ratio (FCR) and feed efficiency ratio (FER) were calculated based on the body weight of the rats using Equations 2 and 3 from Shi et al. (2015).

(2) $$\mathrm{FCR}\;\left(\%\right)=\frac{\text{Feed Intake}}{\text{Body Weight}}$$

(3) $$\mathrm{FER}=\frac{\text{Body weight}}{\text{Feed Intake}}$$

### 2.7 Blood collection

**FIGURE 1:**
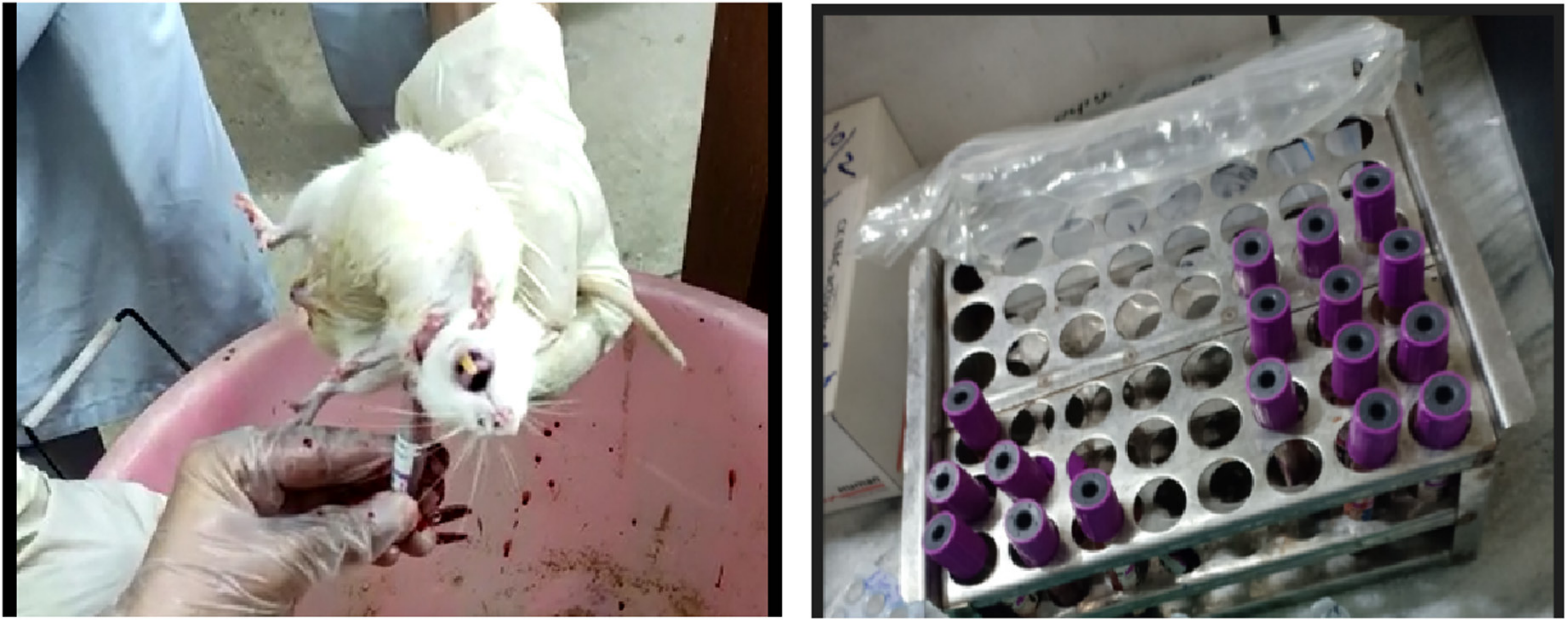
Collection of blood samples.

At the end of the study, anesthesia was administered to all rats before sacrificing them. Using a surgical blade, blood was collected from the neck area and placed in EDTA tubes, as illustrated in Figure 1. The serum was then separated through centrifugation at 5000 rpm and stored at −20°C for subsequent biochemical analysis.

### 2.8 Biochemical analysis

The following factors were measured using commercially available kits and specific methods: low‐density lipid (LDL) (mg/dL) (Cat. No. 10202), serum cholesterol (mg/dL) (Cat. NO. 10028), triglycerides (TAG) (mg/dL) (Cat. No. 10724), serum high‐density lipid (HDL) (mg/dL) (Cat. No. 10017), alanine aminotransferase (ALT) (U/L), aspartate aminotransferase (AST) (U/L), serum bilirubin (mg/dL), alkaline phosphatase (ALP) (U/L), total protein (g/dL), creatinine (mg/dL), blood urea nitrogen (BUN), and uric acid (mg/dL). The Cobas E311 method was used, based on the principle of a spectrophotometer, to determine these parameters. In addition, hemoglobin level (Hb) (g/dL), red blood cell (RBC) count (×10^6^μL), white blood cell (WBC) count (×10^3^μL), monocyte (%), lymphocyte (%), neutrophil (%), and platelet count (×10^5^μL) were determined by Mindary BC‐6200, based on the principles of electrical impedance, flow cytometry, and light scattering, as described by Kulik et al. (2021).

### 2.9 Nitrogen balance

**FIGURE 2:**
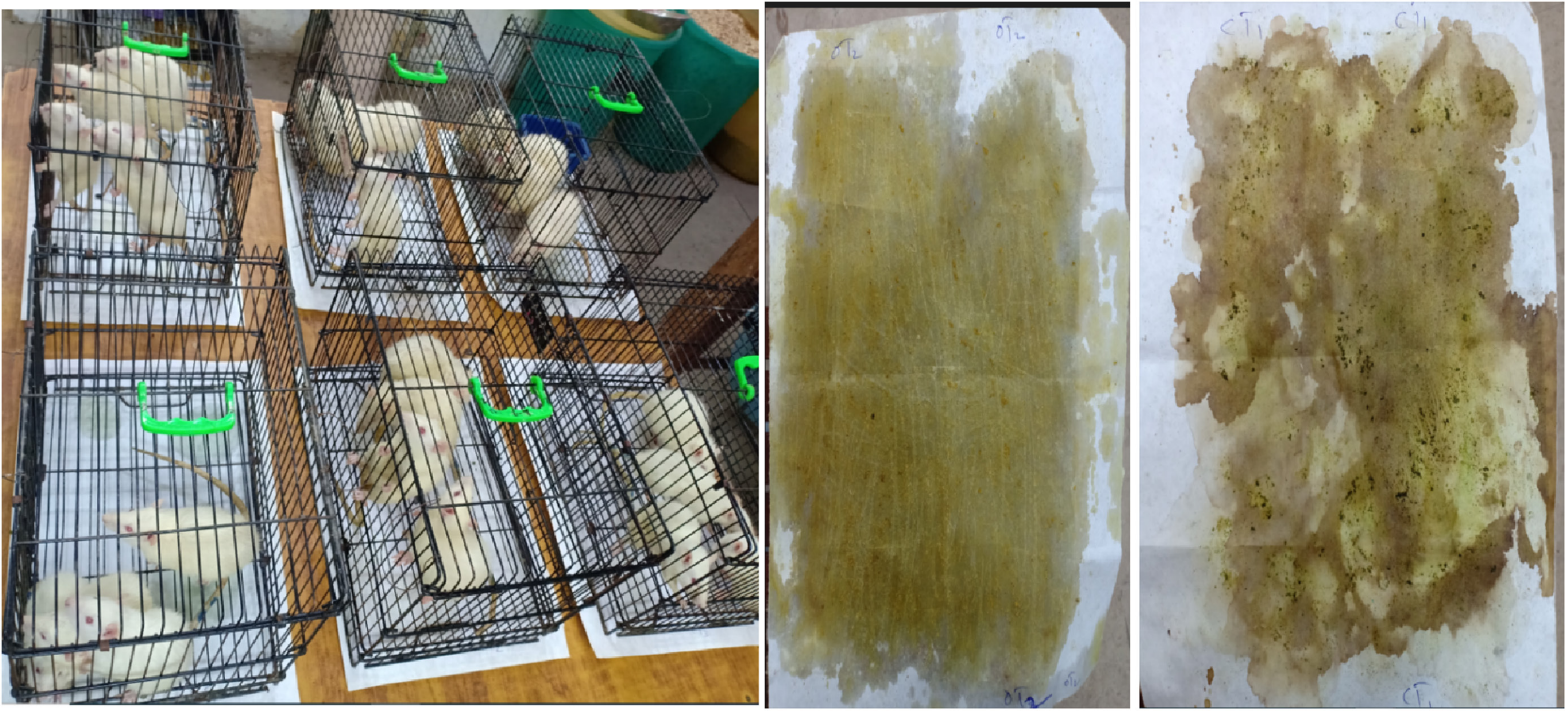
Collection of urine samples for the determination of nitrogen (%).

To collect and determine the nitrogen percentage of urine samples, nitrogen‐free Whatman filter paper was used. Animals were placed in metabolic cages 24 h before the start of the collection period, and pre‐weighted papers were placed underneath the cages on aluminum foil. After 24 h, the papers were collected, weighed, and stored in separate plastic zip‐lock bags for further analysis. Figure 2 shows the collection process for urine. The N% of each paper was determined using the Kjeldahl apparatus, as described by Kumar et al. (2021) and the NRC (2001). The nitrogen balance was estimated by the following equation (4):

(4) $$\text{Nitrogen balance}\;\left(\mathrm{mg}\right)=\left(\text{Nitrogen intake}-\text{Nitrogen in urine}-\text{Nitrogen in feces}\right)$$

### 2.10 Statistical analysis

The data were analyzed using statistical software (Statistics 8.1) with a completely randomized design. Weekly feed intake, water intake, and body weight were analyzed using a two‐way ANOVA. A one‐way ANOVA followed by LSD was used for analyzing other parameters. The level of significance was set at *p* ≤ .05.

### 2.11 Human equivalent dose

Based on our calculations, we determined the optimal human dosage from the most effective amount of onion powder. By taking into account the ratio of human‐to‐rat surface area, we estimate that the equivalent human dose of onion is 181.04 grams with 204 mg of quercetin. Additionally, when factoring in the dry matter content, the recommended dose of onion is 29.19 grams with 220 mg of quercetin.

## 3 RESULTS

### 3.1 Nutrient composition of onion powder and quantification of onion using HPLC‐UV

**FIGURE 3:**
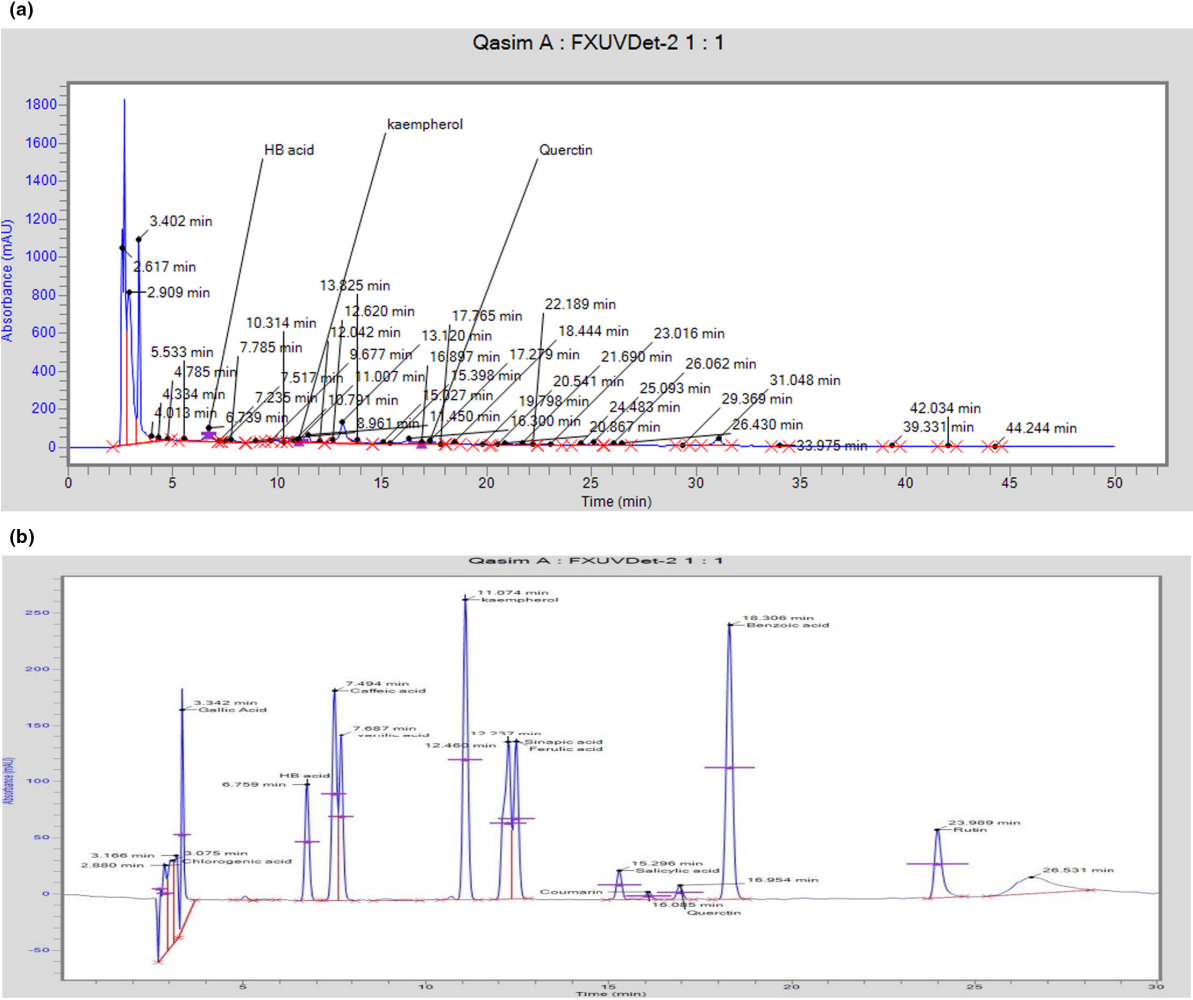
Quantification of quercetin using HPLC‐UV (a) quercetin chromatogram (b) standard.

In an in vivo trial, the nutrient contents of red onions were analyzed. The dry matter (DM) was found to be 16.12%, while crude protein (CP), nitrogen‐free extract (NFE), and crude fiber (CF) were 6.58%, 6.69%, and 1.76%, respectively. The onion also contained a negligible amount of ether extract (EE) (0.82%) and ash (0.27%). The HPLC‐UV analysis revealed the presence of quercetin in onion powder extract, with a peak tR of 16.897 at 360 nm. The quantity of quercetin in onions was measured as 26.69 mg/100 g, compared to the standard quercetin with a value of *t*_R_ observed at 16.954 at 360 nm absorption. The retention factor (k) is a measure of the analyte's retention on a chromatographic column, and the standard K factor is considered as 0.000696. The HPLC‐UV results were presented in the chromatogram, and the peaks are shown in Figure 3a,b.

### 3.2 Effect of onion on feed intake (g)

During the trial, rats that were fed NC had a lower feed intake compared to those that were given treated diets. In the first week, rats that were given OT_2_ and OT_3_ diets had a higher intake compared to those that were given NC and OT_1_ diets. This trend continued in the second week for rats that were given OT_2_ and OT_3_ diets (Table 2).

**TABLE 2:** Effect of onion on feed intake (g).

Treatments	Week 1	Week 2	Week 3
NC	20.66 ± 0.16^b^	21.33 ± 0.12^b^	22.17 ± 0.19^a^
OT_1_	18.23 ± 0.21^b^	20.03 ± 0.23^a^	19.98 ± 0.22^b^
OT_2_	21.25 ± 0.22^a^	21.09 ± 0.09 ^a^	19.62 ± 0.25^b^
OT_3_	21.60 ± 0.09^a^	21.64 ± 0.12^a^	20.98 ± 0.21^b^

Abbreviations: NC, Normal control; OT_1_, Onion treatment 1; OT_2_, Onion treatment 2; OT_3_, Onion treatment 3.

Mean within the same column with different superscripts (a‐d) is statistically different at *p* ≤ .05.

### 3.3 Effect of onion on body weight (g)

During the study, the weight of the rats initially decreased in week 1 of the treatment period. However, by the end of week 3, their weight had increased. In fact, there was a similar trend of weight gain in week 2 compared to week 1. The increase in body weight was significant (*p* ≤ .05) in both week 2 and week 3. Rats that were given OT_2_ (175.36 ± 2.00) and OT_3_ (176.97 ± 1.33) had a higher body weight (*p* ≤ .05) than those fed NC (Table 3).

**TABLE 3:** Effect of different levels of onion on body weight (g).

Treatments	W0	Week 1	Week 2	Week 3
NC	161.06 ± 0.72^c^	156.42 ± 0.53^d^	164.70 ± 1.12^b^	167.01 ± 1.03^a^
OT_1_	160.15 ± 1.19^c^	155.93 ± 1.18^d^	162.94 ± 2.05^b^	166.91 ± 0.02^a^
OT_2_	161.05 ± 0.80^c^	158.42 ± 0.52^d^	166.04 ± 0.79^b^	175.36 ± 2.00^a^
OT_3_	161.12 ± 0.87^c^	164.08 ± 0.74^c^	170.77 ± 0.60^b^	176.97 ± 1.33^a^

Abbreviations: NC, Normal control; OT_1_, Onion treatment 1; OT_2_, Onion treatment: 2 OT_3_, Onion treatment 3.

Mean within the same column with different superscripts (a‐d) is statistically different at *p* ≤ .05.

### 3.4 Effect of onion on water intake (mL)

The rats fed the OT3 diet had the highest water intake within 3 weeks compared to the NC, OT1, and OT2 diets. Water intake in groups OT1, OT2, and OT3 was highest in week 2 compared to the control group (Table 4).

**TABLE 4:** Effect of onion on water intake (mL).

Treatments	Week 1	Week 2	Week 3
NC	24.30 ± 0.16^c^	25.51 ± 0.15^b^	27.23 ± 0.19^a^
OT_1_	27.97 ± 0.21^b^	29.22 ± 0.17^a^	27.42 ± 0.21^b^
OT_2_	27.41 ± 0.14^b^	34.64 ± 0.12^a^	34.04 ± 0.16^a^
OT_3_	37.38 ± 0.13^b^	40.82 ± 0.17^a^	36.38 ± 0.17^c^

Abbreviations: NC, Normal control; OT_1_, Onion treatment 1; OT_2_, onion treatment 2; OT_3_, onion treatment 3.

Mean within the same column with different superscripts (a‐d) is statistically different at *p* ≤ .05.

### 3.5 Effect of onion on nutrient intake (g)

There was no significant difference (*p* ≥ .05) in the intake of crude fiber and ash among all the groups. Rats that were fed the OT1 diet had the highest nutrient intake compared to the other diets. The ether extract in the OT3 diet was the lowest when compared to the NC, OT1, and OT2 diets. There was no significant difference (*p* ≥ .05) in crude protein intake between the NC and OT1 and OT2 and OT3 diets (Table 5).

**TABLE 5:** Effect of onion nutrient intake (g).

Groups	NC	OT_1_	OT_2_	OT_3_
Dry matter	11 ± 0.38^b^	12.31 ± 0.23^a^	11.05 ± 0.22^b^	10.21 ± 0.22^c^
Crude protein	3.05 ± 0.13^a^	3.21 ± 0.17^a^	2.69 ± 0.13^b^	2.47 ± 0.13^b^
Crude fiber	0.33 ± 0.09^a^	0.39 ± 0.11^a^	0.27 ± 0.08^a^	0.26 ± 0.10^a^
Ether extract	1.17 ± 0.07^a^	1.06 ± 0.06^a^	1.05 ± 0.05^a^	0.87 ± 0.08^b^
Ash	0.69 ± 0.11^a^	0.59 ± 0.06^a^	0.51 ± 0.06^a^	0.52 ± 0.05^a^

Abbreviations: NC, Normal control; OT_1_, Onion treatment 1; OT_2_, Onion treatment 2; OT_3_, Onion Treatment 3.

Mean within the same column with different superscripts (a‐d) is statistically different at *p* ≤ .05.

### 3.6 Effect of onion on nutrient digestibility (%)

The rats that were given the OT_2_ diet had the highest recorded levels of dry matter, crude protein, crude fiber, Ether extract, ash, and NFE digestibilities (*p* ≤ .05). However, the levels of crude protein, crude fiber, ash, and NFE digestibility in other rats were not significantly different (*p* ≥ .05). Additionally, NFE digestibility was not significantly different in any of the diets (*p* ≥ .05) (Table 6).

**TABLE 6:** Effect of onion on nutrient digestibility (%).

Nutrient %	NC	OT_1_	OT_2_	OT_3_
Dry matter	82.45 ± 0.08^b^	86.63 ± 0.03^a^	86.35 ± 0.04^a^	86.17 ± 0.02^a^
Crude protein	89.53 ± 0.05^b^	91.20 ± 0.03^a^	90.37 ± 0.04^a^	89.26 ± 0.03^b^
Crude fiber	87.26 ± 0.04^b^	88.84 ± 0.04^a^	89.40 ± 0.06^a^	87.95 ± 0.03^b^
Ether extract	87.91 ± 0.03b^c^	88.84 ± 0.03^b^	89.57 ± 0.06^a^	88.42 ± 0.07b^c^
Ash	86.61 ± 0.04^c^	89.12 ± 0.07^a^	89.59 ± 0.06^a^	88.44 ± 0.06^b^
NFE	96.16 ± 0.06^a^	96.59 ± 0.05^a^	96.88 ± 0.06^a^	96.09 ± 0.04^a^

Abbreviations: NC, Normal control; OT_1_, Onion treatment 1; OT_2_, Onion treatment 2; OT_3_, Onion treatment 3; NFE, Nitrogen free extract.

Mean within the same column with different superscripts (a‐d) is statistically different at *p* ≤ .05.

### 3.7 Effect of onion on feed conversion ratio (%) and feed efficiency ratio

The tests showed a notable improvement (with a significance level of *p* ≤ .05) in the FCR and FER of rats on all the diets tested. Specifically, rats on the OT_3_ and OT_2_ diets had significantly improved FCR (1.18% ± 0.03% and 1.23% ± 0.04%, respectively). Additionally, rats on the OT_2_ and OT_3_ diets had higher FER (0.83 ± 0.04 and 0.83 ± 0.06, respectively) compared to those on the NC and OT_1_ diets (Table 7).

**TABLE 7:** Effect of onion on feed conversion ratio and feed efficiency ratio.

Groups	NC	OT_1_	OT_2_	OT_3_
FCR (%)	2.58 ± 0.05^a^	1.89 ± 0.05^b^	1.23 ± 0.04^c^	1.18 ± 0.03^c^
FER	0.46 ± 0.05^b^	0.49 ± 0.14^b^	0.83 ± 0.04^a^	0.84 ± 0.06^a^

Abbreviations: FCR, Feed conversion ratio; FER, Feed efficiency ratio; NC, Normal control; OT_1_, Onion treatment 1; OT_2_, Onion treatment 2; OT_3_, Onion Treatment 3.

Mean within the same column with different superscripts (a‐d) is statistically different at *p* ≤ .05.

### 3.8 Effect of onion on liver function

Rats that were fed the OT_2_ diet showed a significant improvement in their serum ALT, AST, and total bilirubin levels when compared to those on the NC, OT_1_, and OT_3_ diets. Additionally, rats on the OT_2_ and OT_3_ diets had a significant improvement in their ALP levels (U/L), while no improvement was observed in rats on the OT_1_ and NC diets. A non‐significant (*p* ≤ .05) difference in serum total protein levels was observed across all treatment diets (Table 8).

**TABLE 8:** Effect of onion on liver function.

Groups	NC	OT_1_	OT_2_	OT_3_
ALT (U/L)	228.28 ± 0.29^a^	103.73 ± 0.93^b^	51.84 ± 0.07^d^	81.92 ± 0.91^c^
AST (U/L)	567.53 ± 0.35^a^	193.50 ± 0.49^b^	116.27 ± 0.36^d^	153.40 ± 0.36^c^
Total Bilirubin (mg/dL)	0.24 ± 0.002^b^	0.34 ± 0.06^a^	0.09 ± 0.004^d^	0.11 ± 0.004^c^
ALP (U/L)	541.75 ± 4.03^a^	538.75 ± 5.12^a^	223.75 ± 3.59^b^	226.25 ± 6.39^b^
Total Serum Protein (g/dL)	6.94 ± 0.02^a^	6.78 ± 0.04^a^	6.93 ± 0.04^a^	6.92 ± 0.05^a^

Abbreviations: ALT, Alanine aminotransferase; AST, Aspartate aminotransferase; ALP, Alkaline phosphatase; NC, Normal control; OT_1_, Onion treatment 1; OT_2_, Onion treatment 2; OT_3_, Onion treatment 3.

Mean within the same column with different superscripts (a‐d) is statistically different at *p* ≤ .05.

### 3.9 Effect of onion on renal function and uric acid (mg/dL)

Rats that were fed the OT_2_ diet had a lower BUN level (14.32 ± 0.05 mg/dL) compared to those on the OT_1_ and NC diets. The OT_3_ diet also resulted in a lower BUN level (18.34 ± 0.85 mg/dL), but not as low as the OT_2_ diet. The serum uric acid level was significantly lower (1.12 ± 0.18 mg/dL) in rats on the OT_2_ diet, while there was no significant difference in serum uric acid levels in rats fed the OT_1_ and OT_3_ diets compared to the control. The trend in serum creatinine was not significant, but rats on the OT_2_ diet had a lower creatinine level (0.40 ± 0.05 mg/dL) compared to those on the other onion‐enriched diets (*p* ≤ .05) (Table 9).

**TABLE 9:** Effect of onion on renal function and serum uric acid (mg/dL).

Groups	NC	OT_1_	OT_2_	OT_3_
Creatinine	0.36 ± 0.02^b^	0.49 ± 0.06^a^	0.40 ± 0.05^a^	0.43 ± 0.04^a^
BUN	18.65 ± 0.20^b^	22.78 ± 0.30^a^	14.32 ± 0.05^c^	18.34 ± 0.85^b^
Uric acid	0.92 ± 0.04^c^	1.54 ± 0.09^a^	1.12 ± 0.18^b^	1.40 ± 0.25^ab^

Abbreviations: BUN, Blood urea nitrogen; NC, Normal control; OT_1_, Onion treatment 1; OT_2_, Onion treatment 2; OT_3_, Onion treatment 3.

Mean within the same column with different superscripts (a‐d) is statistically different at *p* ≤ .05.

### 3.10 Effect of onion on lipid profile (mg/dL)

The study found that onion‐enriched diets resulted in lower and higher levels of serum cholesterol, ranging from 58.25 ± 6.07 mg/dL to 79.46 ± 1.11 mg/dL. Compared to the control group, all onion‐treated diets showed a significant decrease in serum cholesterol (*p* ≤ .05). Among the onion‐treated diets, OT3 had the lowest serum LDL levels at 9.32 ± 0.29 mg/dL, while rats fed the NC diet had significantly higher serum LDL levels. Onion‐treated diets OT1 and OT3 had the highest and lowest HDL levels at 54.37 ± 0.35 mg/dL and 29.52 ± 0.38 mg/dL, respectively, compared to the NC diet at 54.08 ± 08 mg/dL (Table 10).

**TABLE 10:** Effect of onion on lipid profile (mg/dL).

Groups	NC	OT_1_	OT_2_	OT_3_
Cholesterol	111.00 ± 5.29^a^	79.46 ± 1.11^b^	72.69 ± 0.57^c^	58.25 ± 6.07^d^
HDL	56.08 ± 0.14^a^	54.37 ± 0.35^b^	39.26 ± 0.84^c^	29.52 ± 0.38^d^
LDL	32.30 ± 0.31^a^	10.55 ± 0.34^c^	14.75 ± 3.30^b^	9.32 ± 0.29^d^
Triglyceride	42.65 ± 0.47^d^	57.77 ± 0.61^c^	60.35 ± 0.20^b^	72.17 ± 1.18^a^

Abbreviations: HDL, High density lipid; LDL, Low density lipid; NC, Normal control; OT_1_, Onion treatment 1; OT_2_, Onion treatment 2; OT_3_, Onion treatment 3.

Mean within the same column with different superscripts (a‐d) is statistically different at *p* ≤ .05.

### 3.11 Effect of onion body immunity and hematology

Rats that were given the OT_3_ diet had a significantly higher WBC count (9.78 ± 0.08 × 103/μL) compared to the rats given the OT_1_ and OT_2_ diets. Neutrophils were found to be higher (42.40 ± 0.12%) in the rats given the OT_1_ diet, followed by the OT_3_ diet (39.49% ± 0.11%), compared to the NC diet (40.60% ± 0.07%). Similarly, onion‐enriched diets OT_3_ and OT_1_ showed a higher trend in monocytes (4.87% ± 0.09% and 4.71% ± 0.09%, respectively) compared to the control diet (2.10% ± 0.06%). The onion‐treated diets had a positive influence on the lymphocyte percentage, with higher percentages (60.31% ± 0.09% and 51.11% ± 0.09%) observed in the OT_2_ and OT_3_ diets compared to the NC diet. A similar trend was seen in the RBC count, which improved in the onion‐offered diets OT_1_ (7.06 ± 0.02 × 106/μL) and OT_2_ (7.07 ± 0.05 × 106/μL), followed by OT_3_ (6.88 ± 0.08 × 106/μL), compared to the NC diet. Similarly, the Hb level (13.55 ± 0.31 g/dL, 12.71 ± 0.37 g/dL, and 11.53 ± 0.08 g/dL) was higher (*p* ≤ .05) in rats given diets OT_2_, OT_1_, and OT_3_, respectively, compared to the NC diet with no onion. The highest platelet count (688.8 ± 6.13 × 105/μl and 672.25 ± 2.75 × 105/μl) was observed in rats given OT_1_ and OT_2_ diets, respectively, compared to the NC diet (512.75 ± 2.98 × 105/μL) (Table 11).

**TABLE 11:** Effect of onion on body immunity and hematology.

Groups	NC	OT_1_	OT_2_	OT_3_
WBC (×10^3^μL)	8.68 ± 0.07^b^	6.62 ± 0.06^c^	6.26 ± 0.04^c^	9.78 ± 0.08^a^
Neutrophil (%)	40.60 ± 0.07^b^	42.40 ± 0.12^a^	34.22 ± 0.10^c^	39.49 ± 0.11^b^
Monocyte (%)	2.10 ± 0.06^b^	4.71 ± 0.09^a^	1.70 ± 0.08^b^	4.87 ± 0.09^a^
Lymphocyte (%)	53.55 ± 0.65^b^	46.52 ± 0.10^d^	60.31 ± 0.09^a^	51.11 ± 0.09^c^
RBC (×10^6^μL)	6.51 ± 0.10^b^	7.07 ± 0.05^a^	7.06 ± 0.02^a^	6.88 ± 0.08^b^
Hb (g/dL)	11.72 ± 0.12^b^	12.71 ± 0.37^a^	13.55 ± 0.31^a^	11.53 ± 0.08^b^
Platelet count (×10^5^/μL)	512.75 ± 2.98^b^	688.8 ± 6.13^a^	672.25 ± 2.75^a^	469.00 ± 3.91^c^

Abbreviations: Hb, Hemoglobin; NC, Normal control; OT_1_, Onion treatment 1; OT_2_, Onion treatment 2; OT_3_, Onion treatment 3; RBC, Red blood cell; WBC, White blood cell.

Mean within the same column with different superscripts (a‐d) is statistically different at *p* ≤ .05.

### 3.12 Effect of onion on nitrogen balance (mg/day)

The study found that rats treated with onions had a positive nitrogen balance, meaning they consumed more nitrogen than they lost from their bodies. The results showed that rats fed OT_1_ and OT_2_ diets had significantly higher nitrogen retention (0.111 ± 0.04 mg/day and 0.085 ± 0.02 mg/day, respectively) compared to those fed OT_3_ and NC diets (Table 12).

**TABLE 12:** Nitrogen balance study.

Parameters mg/day	NC	OT_1_	OT_2_	OT_3_
N Intake	0.451 ± 0.02^b^	0.476 ± 0.03a	0.440 ± 0.10^b^	0.428 ± 0.10^d^
Urinary N	0.027 ± 0.006^c^	0.039 ± 0.008^b^	0.019 ± 0.07^d^	0.050 ± 0.01^a^
Fecal N	0.353 ± 0.008^a^	0.326 ± 0.009^c^	0.336 ± 0.005^c^	0.336 ± 0.009^b^
N Balance	0.071 ± 0.009c	0.111 ± 0.04^a^	0.085 ± 0.02^b^	0.042 ± 0.07^d^

Abbreviations: NC, Normal control; OT_1_, Onion treatment 1; OT_2_, Onion Treatment 2; OT_3_, Onion treatment 3.

Mean within the same column with different superscripts (a‐d) is statistically different at *p* ≤ .05.

## 4 DISCUSSION

Parallel to our research, Bhattacharjee et al. (2013) reported on the approximate composition of red onions from Bangladesh and India. In contrast to the other study, which revealed higher DM (17.23%) and lower CP (1.48%), this study showed lower DM (16.12%) and higher CP (6.58%). Comparing this study's ether extract (EE) (0.82%), ash (0.27%), and CF (1.76%) results to the other study's EE (0.721%), ash (1.659%), and CF (1.659%) results, there was hardly any difference. This diversity may result from the different types of onions, the location, and local environmental factors. With a peak *t*_*R*_ of 16.897 at 360 nm, our HPLC‐UV results demonstrated the existence of quercetin in onion powder and demonstrated that red onions contain a quercetin concentration of 26.69 mg/100 g. Kwak et al. (2017) used HPLC analysis to measure the levels of quercetin in several onion cultivars, including red onion, yellow onion, and chartreuse onion. They freeze‐dried the onion samples at −70°C for 48 h, and they found that the red onion's outer shell has a quercetin level of 0.59 mg/g dry weight. Additionally, how an onion is grown in various places may affect this. Elberry et al. (2014) found that the methanolic extract of red onion scales had 60.1 mg/g of dry quercetin, compared to the typical quercetin concentration of 1.5 mg/mL. Among the 28 vegetables and 9 fruits, onion had the greatest quercetin level, which is relevant to the study by Hertog et al. (1992). In a similar vein, Gorinstein et al. (2008) confirmed that red onions have a quercetin level that is 14 times higher than that of garlic and two times higher than that of white onions.

Based on the results shown in Table 2, rats that were given OT_2_ and OT_3_ diets had higher food intake during the weeks 1 and 2 compared to those given NC and OT_1_ diets. This could be due to the increase in digestibility and fiber content of the diet. When onion is heated, its sulfur compounds degrade into bis‐propenyl disulfide, which gives it a sweet taste and flavor (Gonzalez et al., 2010). This could be why onions were dried in a furnace or oven. In a study conducted by González‐Peña et al. (2014), rats that were offered a diet with 10% onion showed an improvement in food intake. Another study showed that onion extract offered to Torki Qashqai suckling lambs at the levels of 150 mg/kg and 250 mg/kg enhanced the ghrelin hormone, which stimulates appetite and is considered a good feed additive for ruminant nutrition (Amiri et al., 2019). Onion was also observed to increase gustatory peristalsis movements when added to the diet (Lucinda A et al., 2004). In common carp (Cyprinus carpio), better food intake was observed upon supplementing the diet with quercetin at 200 and 800 mg/kg (Ghafarifarsani et al., 2022). In a study conducted on Vencob‐400 strain broilers, quercetin offered at the level of 1 g/kg/bw had a promising effect on food intake in broiler chicks. This could be attributed to quercetin's antioxidant and hypolipidemic activity, which protects cells from free radicals and lowers lipid levels by influencing lipid metabolism in the liver (Parmar et al., 2020).

Based on the findings presented in Table 3, there was a significant increase in body weight (*p* ≤ .05) during both weeks 2 and 3. Rats that were given OT_2_ (175.36 ± 2.00 g) and OT_3_ (176.97 ± 1.33 g) had a higher body weight (*p* ≤ .05) compared to those that were fed NC. Goodarzi et al. (2014) also noticed an increase in body weight in broiler chicks when they were given a diet containing 3% onion bulbs. Another study conducted on broiler chicks by Aji et al. (2011) and Sohaib et al. (2015) also observed a gain in body weight when different levels of onion and garlic were included in their diet. This positive effect is believed to be due to the presence of quercetin and sulfur compounds in the onion, which have properties such as free radical scavenging, antibacterial, and improved digestion and absorption.

During week 2, the water intake of groups OT_1_, OT_2_, and OT_3_ was the highest compared to the control group, according to Table 4. This increase in water intake could be attributed to a rise in feed intake and improved fiber digestibility. A study by Aji et al. (2011) reported that the increase in water intake seen in diets containing onions may be due to the high fiber and sulfur content in onions, which stimulate the thirst center in the brain. The fiber content in the feed and onion may also require more water for digestion and absorption, leading to the observed increase in water intake.

Higher nutrient intake among diets may improve due to the increase in feed intake and digestibility. Rats fed an OT_1_ diet consumed higher nutrients than the other groups, according to (Table 5). This might be a result of the onion being more readily available in the OT_1_ diet, and rats consumed more onion as a result of the greater onion‐to‐feed ratio. A higher DM intake was observed due to the higher intake of fiber and feed. Other levels did not show an increase in DM intake because some anti‐quality compounds in the onion and feed, such as tannin and alkaloids, may decrease the intake. Higher CP intake was observed in rats fed an OT_1_ diet because nitrogen balance was seen as positive in OT_1_ and more protein was utilized in the muscles of the body. A previous study reported that onion extract (0, 1, and 2%) offered in diets facilitates the intake of protein due to sulfur‐containing amino acids and flavonoids (Kim, 2007). Increased fiber intake might be due to an increase in feed intake and the release of the ghrelin hormone, which stimulates the appetite. Onion also contains non‐digestible fructooligosaccharides (Inulin). Prebiotics are non‐digestible food ingredients that are selectively fermented by the gut microbiota, promote the growth of healthy microbes, and improve digestion, absorption, and immune response. It has been concluded that prebiotics facilitate the digestion of fiber by enhancing bacterial activity in the gut. Bacteria in the gut break down the fiber (Bouhnik et al., 2004; Gibson, 1998; Rahim et al., 2021). Research has shown that prebiotics can enhance the digestion of fiber by modulating the gut microbiota. Supplementing the diet with prebiotics (fructooligosaccharides and inulin) can increase the synthesis of short‐chain fatty acids (SCFAs) in the colon, which are the end product of fiber fermentation by gut bacteria. It is concluded that prebiotics facilitate the digestion of fiber by enhancing microbe's activity in the gut.

In a recent study, rats fed diets containing onions showed significant improvements in the digestibility of dry matter, crude protein, crude fiber, ether extract, ash, and NFE compared to the control group. Among the onion‐fed rats, those on the OT_2_ diet had the highest levels of digestibility (*p* ≤ .05) (Table 6). The improvement in digestibility may be due to certain antinutritional compounds in onions that interfere with protein enzyme availability for digestion (Wanapat et al., 2014). Onion consumption may also increase the release of the ghrelin hormone, stimulating appetite and improving DM digestibility (Amiri et al., 2019). Previous studies have shown that friendly bacteria, rumen microbiota activity and retention time, and the availability of free amino acids in muscles can all contribute to improved DM digestibility (Ahmed et al., 2009; Aiad, 2005; Gupta et al., 2005; Saleh et al., 2015; Sarwar & Khan, 2004a, 2004b). Onion also contains quercetin and kaempferol, important flavonoids with antioxidant and free radical scavenging activity, which can improve protein digestion by increasing the availability of free amino acids in muscles (Saleh et al., 2015). However, ingestible complexes with nutrients and the lowered activity of digestive enzymes can restrict protein digestibility in some cases (Świeca et al., 2013). Onion can enhance ether extract (EE) digestibility by releasing pancreatic enzymes and increasing the activity of enzymes important for lipid digestion in the liver (FrAnKIČ et al., 2009; Mirzaei‐Aghsaghali, 2012). Phenols, polyphenols, terpenoids, polypeptides, lectins, and alkalis found in onions can also improve the digestibility of lipids by enhancing digestive enzyme activity (Cross et al., 2007). Microbes in the rumen can play a crucial role in fiber digestibility by fermenting fiber and producing short‐chain fatty acids (SCFAs) that provide energy to the body (Nisa et al., 2006; Sarwar & Khan, 2004a; 2004b). However, garlic essential oil has no significant effect on the digestibility of dry matter and fiber (Busquet et al., 2005). Studies have shown that quercetin can improve nutrient digestibility in Korean local goats (Cho et al., 2010; Cho et al., 2017). Increased activity of starch digestive enzymes has also been linked to improved carbohydrate digestibility.

Based on the data presented in Table 7, it was observed that rats that were fed with OT_2_ and OT_3_ diets showed a significant improvement in both FCR and FER. This can be attributed to the fact that the rats had a higher feed intake, gained more body weight, and were able to utilize the feed more efficiently. However, rats that were fed with OT_1_ diet did not show any improvement in FCR and FER due to lower feed intake, leading to less body weight gain. These findings are consistent with the study conducted by Omar et al. (2020), where onion extract was added to the diet at different concentrations (1, 2, and 3 g/kg), and it was found that the levels of 1 and 2 g/kg showed a positive impact on FCR by promoting gut health, improving amino acid digestion, and increasing the intestinal surface area for absorption. Onion also has the ability to prevent the growth of harmful pathogens and enhance the digestion of amino acids. However, the study conducted by Liu et al. (2013) did not observe any improvement in FCR values after adding quercetin, while Liu et al. (2014) found that FCR improved after administering 0.2 and 0.4 g/kg of quercetin to laying hens. This could be due to the antibacterial and anti‐inflammatory properties of quercetin. Additionally, Bang et al. (2009) and Lee et al. (2008) both reported positive effects of onion and quercetin on FER values.

The rats that were given the OT_2_ diet showed a significant improvement in their serum ALT, AST, and total bilirubin levels as compared to those given the NC, OT_1_, and OT_3_ diets. As per Table 8, liver enzymes and bilirubin levels were reduced in rats that were given the OT_2_ and OT_3_ diets with higher quercetin content. A study by Rana et al. (1996) discovered that onion has a protective effect on liver cells due to the presence of functional compounds such as flavonoids, quercetin, and sulfur‐containing compounds. These compounds lower the liver enzyme level by safeguarding the liver cells against oxidative stress and inflammation. Bors and Saran (1987) reported that onions have free radical scavenging activity. Zhang et al. (2006) also highlighted the positive effect of quercetin in mice against iron overload toxicity in the liver cells. Similarly, another study reported by Afanas' ev et al. (1989) discovered a decrease in MDA and lipid peroxidation in liver cells. Quercetin also restored the level of enzymes by promoting the health of the individual, as reported by de David et al. (2011) and Kumar Mishra et al. (2013). Significantly higher serum total protein was observed in rats offered OT_2_ and OT_3_ diets; this might be due to the feed's high onion and quercetin content. Improved protein status has been linked with liver health; quercetin and onion prevent the liver from oxidative stress (Karakilcik et al., 2004).

The levels of serum BUN and creatinine in the body are dependent on the proper functioning of the kidneys. When the kidneys are compromised, they are unable to excrete BUN and creatinine, causing their levels to rise. Onions contain fiber that acts as a prebiotic, giving strength to microbes to use urea nitrogen as a nitrogen source to synthesize protein, resulting in a decrease in BUN. Rats fed the OT_2_ diet had lower BUN levels (14.32 ± 0.05 mg/dL) compared to those on the OT_1_ and NC diets. In addition, rats on the OT_2_ diet had significantly lower serum uric acid levels (1.12 ± 0.18 mg/dL) than those on the OT_1_ and OT_3_ diets, while there was no significant difference in serum uric acid levels in rats fed the OT_1_ and OT_3_ diets compared to the control. The oxidation of amino acids and nitrogen produces ammonia, which is toxic for cells and is excreted from the kidneys in the form of urea. Onion and quercetin significantly protect the renal morphology and decrease the levels of BUN and creatinine, as shown in Table 9. Haidari et al. (2008) reported the renal protective effect of onion and quercetin in rats induced with potassium oxonate. Quercetin reduces lipid peroxidation and inflammation in the glomerular membrane of the kidney (Coskun et al., 2005), while another study showed that quercetin reduces free radical formation in gentamicin GM‐induced kidney injury (Abdel‐Raheem et al., 2009). The synthesis of uric acid involves xanthine dehydrogenase, which is converted into xanthine oxidase. Xanthine oxidase catalyzes hypoxanthine into xanthine, resulting in the production of reactive oxygen species (ROS). Onion and quercetin combat free radicals and restore the functioning of the kidneys (Noone & Marks, 2013; Saleh et al., 2017). Uric acid, BUN, and creatinine were reduced in rats fed the OT_2_ diet compared to treatment diets. This may be due to less production of ROS and more binding of quercetin to xanthine oxidase, leading to lower uric acid synthesis (Neogi et al., 2012). OT_1_ and OT_3_ diets did not show an improvement in serum uric acid, which may be due to the binding of quercetin with anti‐quality compounds in onions such as saponins, phytic acid, and tannins. Quercetin reversed cadmium‐induced renal lipid peroxidation and reduced uric acid levels (Wang et al., 2012).

In Table 10, it was observed that rats fed onion‐treated diets had significantly decreased levels of total cholesterol and LDL. All onion‐treated diets showed a significant reduction in serum cholesterol (*p* ≤ .05) compared to the control group. Among the onion‐treated diets, the diet with the highest onion concentration (OT3) resulted in the lowest serum LDL levels at 9.32 ± 0.29 mg/dL, while the rats fed the NC diet had significantly higher serum LDL levels. This could be due to increased lipid digestibility and a restriction in the HMG‐CoA reductase activity in the liver, which is crucial for lipid synthesis and cholesterol absorption in the intestine (Hasimun et al., 2011). The decreased lipid levels were attributed to the increased digestibility of fat and fiber. Moreover, the fiber content in the onion and diet helped to prevent cardiovascular problems by lowering blood glucose and lipid levels. In a study by Babu and Srinivasan (1997), it was found that offering 3% onion powder decreased cholesterol, lipid peroxidation, and triglyceride levels. Kim (2007) reported similar results, observing the lipid‐lowering effect of onion skin on hyperlipidemia‐induced mice. Onion helps to enhance the release of bile acids for the synthesis and absorption of fatty acids and stimulates the pancreatic enzymes. Sebastian et al. (1979) observed that quercetin and sulfur compounds in onions lower lipid synthesis by oxidizing free‐available thiol compounds. Quercetin was also found to attenuate lipid levels (Qureshi et al., 2011). However, in this study, serum HDL was not increased in rats fed onion‐enriched diets, leading to an increase in triglyceride levels. HDL's function is to transport bad cholesterol from the arteries into the liver for metabolism and excretion.

Based on the data presented in Table 11, it appears that diets including onions may have a positive impact on body immunity and hematological values. This could be due to a variety of factors, such as the high protein content in the diet, improved liver function, increased digestibility of protein, and the anti‐inflammatory and antioxidant properties of onions and quercetin. Rats fed the OT3 diet showed a significant increase in their white blood cell count (WBC), which may be attributed to the high quercetin content and increased feed intake. WBCs, or leukocytes, play an important role in fighting harmful and foreign substances by utilizing mechanisms like phagocytosis, antibody production, and the release of chemical mediators. The percentage of neutrophils, monocytes, and lymphocytes also improved in onion‐treated diets compared to the control. This is likely due to the anti‐inflammatory and antioxidant properties of quercetin and sulfur compounds found in onions, which support these types of cells. Previous studies, such as Akrami et al. (2013), have observed positive effects on WBC and RBC counts with the addition of onion powder due to its immunomodulatory response. Flavonoids, like quercetin, have been shown to enhance the release of erythropoietin, which stimulates stem cells to produce more red blood cells. Additionally, quercetin has been shown to enhance immune responses in lymphoid organs, as noted by Manach et al. (2004) and Selvakumar et al. (2013). However, Ohlsson and Aher (2014) found lower RBC counts and Hb levels in rats fed the OT3 diet, which could be attributed to the presence of disulfide compounds in onions that produce H_2_O_2_ in the presence of Hb and glutathione S‐transferase within the intact erythrocytes. High levels of onion intake have also been linked to adverse effects such as hemolytic anemia, decreased pack cell volume, hemoglobin level, and red blood cell count, as observed by Oboh (2004). On the other hand, Munday et al. (2003) found significant improvements in platelet count in rats fed onion diets compared to the control.

Rats fed the OT_1_ and OT_2_ diets showed significantly positive nitrogen balance, which may be related to the biological effects of quercetin, enhanced protein digestibility, and higher CP intake (Table 12). According to the findings, rats fed the OT_1_ and OT_2_ diets retained nitrogen at significantly higher rates (0.1110.04 mg/day and 0.0850.02 mg/day, respectively) than rats fed the OT3 and NC diets. Maintaining a healthy liver is essential for properly metabolizing protein and maximizing its absorption by the body. The antioxidant enzymes in the body are increased, as are the amounts of amino acids in the liver, by quercetin and onion, both of which have free radical scavenging properties. Cho et al. (2010) provided Korean native goats with quercetin at varied concentrations (0, 500, and 1000 ppm) by varying the amount of concentrate and roughage (RC ratio). Due to the antioxidant and anti‐inflammatory properties of quercetin, the addition of 500 ppm showed a good nitrogen balance. The antioxidant, anti‐inflammatory, and increased enzyme activity of onions regulate the nitrogen balance. They also balance the body's production and use of compounds containing nitrogen, as well as the activity of gut microbes that are crucial to fermentation and nitrogen cycling. Onion contains substances rich in sulfur, including glutathione, an antioxidant that prevents free radicals from causing cell damage. Refer to Cardozo et al.'s (2004) research, which discovered that the onion inhibits the deamination that may be associated with a rise in peptide and AA‐N (Amino Acid Nitrogen) concentrations. The essential oils in onions and garlic increase the availability of protein in the gut and lower the ruminal NH3‐N concentration. As a result, ruminants were able to have greater amino acid availability in the small intestine when protein deamination stopped in the rumen. Rats fed an OT_3_ diet did not exhibit a positive nitrogen balance, which is relevant to this investigation. This might be caused by antinutritional chemicals in onions that prevent protein enzymes from being available and active. Probiotic lactic acid bacteria help in digestion by acting as an aid. Due to the presence of fermentable carbohydrates such as fructoligosaccharides and inulin, onions also function as prebiotics (Sarwar & Khan, 2003). This study found that the gut's pH plays a critical function and that a drop in pH causes free NH_3_ to change to an ionic form (NH_4_). Ammonia in its ionic state (NH_4_) is highly reactive, forms bonds with fibrous substances, and cannot be expelled from the body. This is how fermentable carbs improve N retention. In the same proportion, higher N retention was seen in the ammoniated straw diet when a significant amount of free NH_3_ had been fixed by the fermentable carbohydrates (Sarwar & Khan, 2004a; 2004b). Onions also contain soluble fiber.

## 5 CONCLUSION

Onion significantly improved the uric acid levels and had an improved effect on the feed, water, nutrient intake, nutrient digestibility, nitrogen balance, biochemical, and hematological parameters. Consuming red onions in meals, salads, and sandwiches can help a healthy individual have lower uric acid levels.

## AUTHOR CONTRIBUTIONS

**Muhammad Umer:** Conceptualization (equal); formal analysis (equal); methodology (equal); writing – original draft (equal). **Mahr un Nisa:** Conceptualization (equal); supervision (equal). **Nazir Ahmad:** Writing – original draft (supporting). **Muhammad Abdul Rahim:** Validation (equal). **Ladislaus Manaku Kasankala:** Writing – review and editing (equal).

## FUNDING INFORMATION

No funding.

## CONFLICT OF INTEREST STATEMENT

All authors have no conflicts of interest.

## ETHICS STATEMENT

The experimental procedure was approved by the Animal Ethical Committee of Government College University, Faisalabad, Punjab, Pakistan.

## CONSENT FOR PUBLICATION

All authors agreed to the publication of this manuscript.

## Data Availability

Even though adequate data have been given in the form of tables, all authors declare that if more data are required, the data will be provided on a request basis.
